# Improved Immune Response to the Third COVID-19 mRNA Vaccine Dose in Hemodialysis Patients

**DOI:** 10.1016/j.ekir.2022.09.005

**Published:** 2022-09-11

**Authors:** Daisuke Kanai, Hiromichi Wakui, Tatsuya Haze, Kengo Azushima, Sho Kinguchi, Tomohiko Kanaoka, Yoshiyuki Toya, Nobuhito Hirawa, Hideaki Kato, Kazushi Uneda, Fumimasa Watanabe, Kanako Hanaoka, Masaaki Hanaoka, Hiroshi Mitsuhashi, Satoshi Yamaguchi, Toshimasa Ohnishi, Kouichi Tamura

**Affiliations:** 1 Department of Medical Science and Cardiorenal Medicine, Yokohama City University Graduate School of Medicine, Yokohama, Japan; 2 Center for Nobel and Exploratory Clinical Trials (Y-NEXT), Yokohama City University, Yokohama, Japan; 3 Department of Nephrology and Hypertension, Yokohama City University Medical Center, Yokohama, Japan; 4 Infection Prevention and Control Department, Yokohama City University Hospital, Yokohama, Japan; 5 Department of Kampo Medicine, Aizu Medical Center, Fukushima Medical University School of Medicine, Aizuwakamatsu, Japan; 6 Kohsaikai Kamioooka Jinsei Clinic, Yokohama, Japan; 7 Kohsaikai Yokohama Jinsei Hospital, Yokohama, Japan

**Keywords:** antibody, BNT162b2, dialysis, spike protein SARS-CoV-2, vaccine

## Introduction

A third dose of SARS-CoV-2 mRNA vaccine has been administered in several countries.^1^^,^^2^ Patients with end-stage kidney disease have a weak immune response to 2-sessional doses of the mRNA vaccine.^3^, ^4^, ^5^, ^6^, ^7^ Because patients undergoing hemodialysis (HD) are at a high risk for COVID-19 severity and death, they have been prioritized in vaccination programs worldwide.^8^^,^^9^

To the best of our knowledge, there are no reports comparing humoral immunity after the third vaccination in hundreds of patients undergoing HD with that of healthy controls. However, the existing studies are limited by their small sample size. Therefore, in this multi-institutional retrospective study, we examined the impact of the BNT162b2 (Comirnaty, Pfizer-BioNTech) vaccine third dose on anti-SARS-CoV-2 spike protein S1 IgG antibody (anti-S IgG) titers in patients on HD and health care workers (HCWs).

## Results

**Figure 1:**
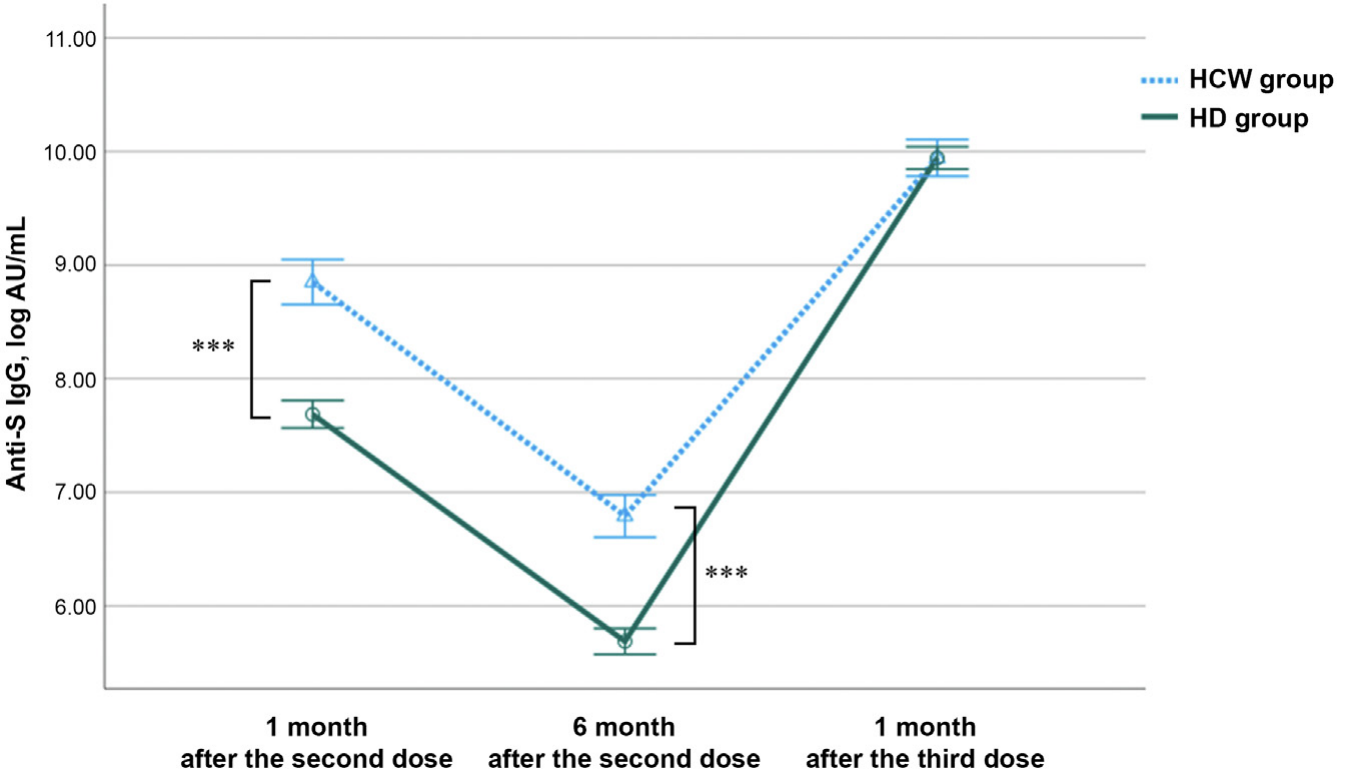
Changes in the anti-S IgG titers. Changes in the natural logarithmic form of the anti-S IgG titers are shown. Dots and error bars show mean ± 95% confidential interval of log-transformed anti-S IgG titers in the HD group (circular dots) and in the control HCW group (triangular dots). HD group versus HCW group via Welch’s *t*-test. ∗∗∗*P* < 0.001. HD, hemodialysis; HCW, health care worker.

**Figure 2:**
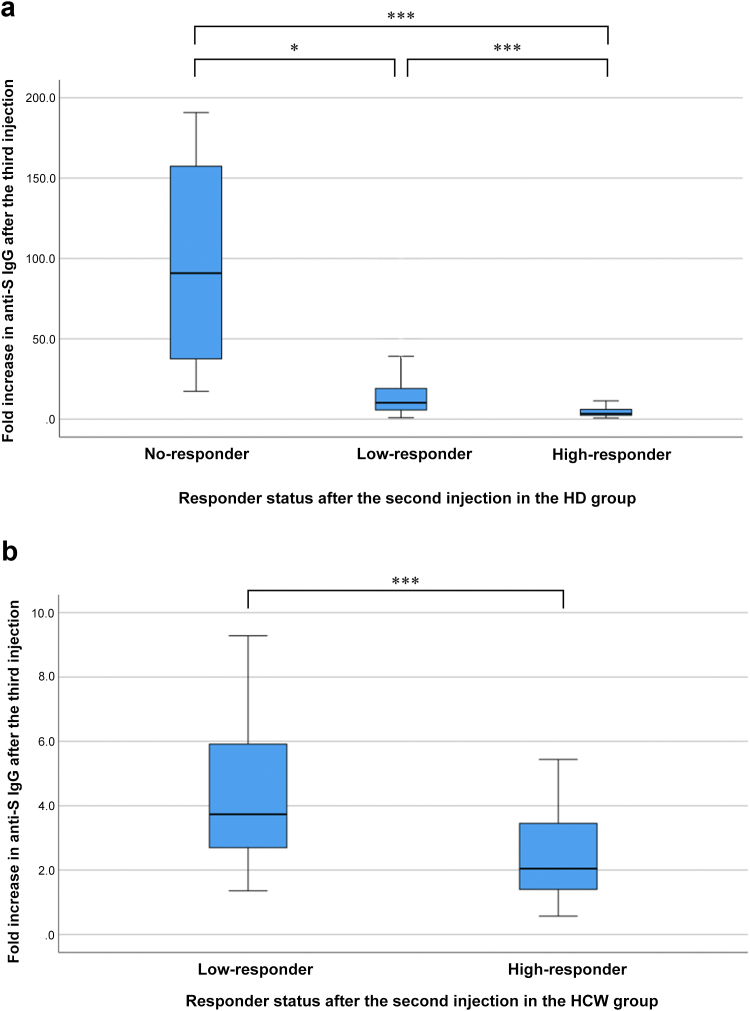
Fold increase in the anti-S IgG titer after the third dose in each responder status group. (a) The box and whisker chart shows a fold increase in the anti-S IgG titer after the third dose in each responder status group compared with that after the second dose in the HD group. The whisker on the upper side is the maximum in the nonresponder group and is drawn to 1.5 times the interquartile range larger than the third quartile in the low/high responder groups. The whisker on the lower side is the minimum. The Kruskal–Wallis test was performed to compare all 3 groups. A *post hoc* analysis was performed using the Bonferroni test to compare 2 groups. ∗*P* < 0.05, ∗∗*P* < 0.01, ∗∗∗*P* < 0.001. (b) The box and whisker chart shows a fold increase in the anti-S IgG titer after the third dose in each responder status group compared with that after the second dose in the HCW group. The whiskers on the upper side are drawn to 1.5 times the interquartile range larger than the third quartile and the ones on the lower side are minimum. The Mann–Whitney *U*-test was performed to compare the low responder and high responder groups. ∗∗∗*P* < 0.001. HCW, health care worker; HD, hemodialysis.

The final analyses included 350 patients on HD and 130 HCWs (Supplementary Table S1). After the second vaccination, the patients undergoing HD had significantly lower anti-S IgG titers than the HCWs with a median of 2538.8 (interquartile range [IQR]: 1185.6–4938.1) AU/ml versus 7645.1 (IQR: 4856.8–11,000) AU/ml 1 month after the second dose (*P* < 0.001); and 312.8 (IQR: 157.9–613.6) versus 803.8 (IQR: 498.4–1342.7) AU/ml at 6 months after the second dose (*P* < 0.001), respectively (Supplementary Table S2). Nevertheless, the increased titers 1 month after the third dose were comparable in both groups with a median of 24,500 (IQR: 11,000–40,000) AU/ml in the patients on HD versus 20,000 (IQR: 12,750–32,250) AU/ml in the HCWs. Similarly, the log-transformed anti-S IgG level in the HD group was significantly lower than that in the HCWs from 1 to 6 months after the second vaccination (Figure 1). The third dose of BNT162b2 increased anti-S IgG titers of the patients undergoing HD toward the same level as that of the HCWs with a mean of 9.94 (95% confidence interval: 9.84–10.05) log (AU/ml) in the HD group versus 9.94 (95% confidence interval: 9.82–10.07) in the HCW group (Figure 1). Although the seronegativity rate at 6 months after the second dose was significantly higher in the HD group (7.7%, *n* = 27) than that in the HCW group (0%, *n* = 0; *P* < 0.001), the seronegativity rates at 1 month after the third dose were 0% in both groups (Supplementary Table S3). The rate of high responders was 48 of 350 (13.7%) in the HD group versus 73 of 130 (56.2%) in the HCW group at 1 month after the second dose and 306 of 350 (87.4%) versus 125 of 130 (96.2%) 1 month after the third dose (Supplementary Tables S4 and S5). In addition, after the third dose, no responders or low responders had a significant increase in anti-S IgG titers compared with that in high responders with a 90.8-fold (IQR: 27.5–174.1) increase in no responders, a 10.2-fold (IQR: 5.7–19.2) increase in low responders, and a 3.3-fold (IQR: 2.4–6.1) increase in high responders (Figure 2a). Similarly, after the third dose in the HCW group, a greater fold increase in anti-S IgG titers was observed in low responders than that in high responders (3.7-fold [IQR: 2.7–6.0] vs. 2.0-fold [IQR: 1.4–3.5]; *P* < 0.001; Figure 2b).

## Discussion

To the best of our knowledge, this is the first report comparing the anti-S IgG titers of patients on HD with those of HCWs after the third dose of the BNT162b2 vaccine with hundreds of participants.

Patients with end-stage kidney disease have an impaired humoral response to the vaccine antigen due to the deleterious effect of uremic toxins on the generation of antigen-specific T follicular helper cells, B cells, and plasmacytes.S1–S4 Kt/V_urea_ is positively correlates with serological response after the second dose of COVID-19 mRNA vaccines in patients receiving HD.^6^ Hemodiafiltration has better middle molecules weight toxin clearance than HD.S5 Hemodiafiltration is reported to have greater effect on maintaining humoral immunity after influenza vaccination than HD.S6 To date, it is unclear that hemodiafiltration has better influence on humoral response to COVID-19 vaccines. In the present study, there was no significant difference in anti-S IgG titers between patients on HD and those on hemodiafiltration at any measured point (data not shown). The peak levels of anti-S IgG titers in HD patients after the second dose are approximately one-ninth to one-third of those in healthy individuals,^3^^,^^6^^,^^7^^,^S7,S8 and the antibody titers wane over time: the titers at 6 months after the second dose decrease to approximately 10% of their peak levels in both groups.^7^ Waning humoral immunity increases the risk of hospitalization or death due to COVID-19.S9 Therefore, it is essential to raise the antibody titer especially in immunocompromised patients like patients with end-stage kidney disease. A third dose of BNT162b2 or mRNA-1273 (Moderna) in patients with end-stage kidney disease improves their cellular and humoral immunity.S10 In this study, the anti-S IgG titers in the HD group remained significantly lower than those in the HCW group for 6 months after the second dose and were 40% of those in the HCW group just before the third dose. Notably, after the third dose, the titers in the HD group increased to a similar level to that in the HCW group.

Based on the responsiveness to the SARS-CoV-2 mRNA vaccination, Espi *et al.*S11 proposed classification of responses into 3 categories (no, low, and high response) using the postvaccination anti-S IgG antibody titer values. They found a significantly strong positive correlation between the anti-S IgG titer (measured using a chemiluminescence assay) and the neutralizing capacity of the serum and established that a titer ≥997 BAU/ml (equivalent to 7021 AU/ml in our study) was associated with the viral neutralizing capacity of the serum.S11 Using this threshold, they defined patients with anti-S IgG titer ≥997 BAU/ml as high responders to the vaccine.S11 In our study, the rate of no responders or low responders in the HD group was 2 times higher than that in the HCW group after the second vaccination. Nevertheless, 86.8% of no responders or low responders in the HD group experienced a higher increase in response status after the third dose. These results suggest that the conventional 2-sessional dose scheme with the BNT162b2 vaccine provided insufficient protective humoral immunity against COVID-19 for patients undergoing HD who are naïve of SARS-CoV-2 infection.

In addition, we examined the anti-S IgG titer fold change in response to the third vaccination of each responder 1 month after the second dose. Interestingly, in both groups, the lower the response to the second dose, the greater the anti-S IgG titer fold increase in response to the third dose. Therefore, a population with no or low immune response to the second dose should be administered the third dose.

We speculated that 3 doses of the BNT162b2 vaccine might hit a “ceiling of humoral immunity,” and the median ceiling titer would be approximately 25,000 AU/ml. This hypothesis is consistent with the results of this study as follows: (i) the anti-S IgG titers in both groups were similar after the third dose and (ii) participants with a higher humoral response to the second dose had a smaller increase in the anti-S IgG titers after the third dose. In other studies, the anti-S IgG titers of high responders on HD and of participants not on HD were similar at median 20,000–25,000 AU/ml after the third dose of BNT162b2.S12–S14 In addition, a fourth dose of BNT162b2 for healthy young people induced only a slight increase in the anti-S IgG titer, that is, a 1.3-fold increase after the fourth dose versus a 2.2-fold increase after the third dose.S15

Our study has some limitations. First, it was a retrospective study. Second, the participants’ backgrounds differed significantly between the groups. Third, only adult patients undergoing HD (not less than 20 years old) were included. Fourth, it was difficult to exclude asymptomatic participants who were infected with SARS-CoV-2. Fifth, we examined the anti-S IgG titers but no other neutralizing antibodies or cellular immunity. Sixth, the only vaccine investigated was BNT162b2 (Pfizer-BioNTech); patients who received other mRNA vaccines were not included.

In conclusion, this study revealed that a third dose of the BNT162b2 vaccine was effective for increasing anti-S IgG titers in patients undergoing HD at the same level as that in the general population. In addition, it boosted the titers more in no responders or low responders than in high responders. A third dose of the BNT162b2 vaccine is strongly recommended for patients with no response or low response to the 2-session vaccine protocol.

## Disclosure

All the authors declared no conflict of interest.

## Supplementary Material

**Supplementary Methods.**

**Supplementary Results.**

**Supplementary References.**

**Table S1.** Characteristics of participants at baseline.

**Table S2.** Titers of anti-S IgG after the second and third vaccination doses.

**Table S3.** The seronegativity rate of SARS-CoV-2 spike protein antibody after the second dose.

**Table S4.** Number of each responder status group between the second and third doses of BNT162b2 in the HD group.

**Table S5.** Number of each responder status group between the second and third doses of BNT162b2 in the HCW group.
