# Supplementary material for: Improved Immune Response to the Third COVID-19 mRNA Vaccine Dose in Hemodialysis Patients
Source: Kidney Int Rep. 2022 Sep 11;7(12):2718–21. doi: 10.1016/j.ekir.2022.09.005 (PMC9464310; doi:10.1016/j.ekir.2022.09.005)
Supplement: Supplementary File (PDF) [file mmc1.pdf]

Supplementary Materials

Supplementary Methods

Study design

This retrospective study included data from four dialysis facilities (Kamioooka Jinsei Clinic, Yokohama, Japan; Bunko Jin Clinic, Yokohama; Kanazawa Clinic, Yokohama; and Oppama Jinsei Clinic, Yokosuka, Japan), which are affiliated dialysis centers of Yokohama City University Hospital and Yokohama City University Medical Center, Yokohama, Kanagawa, Japan. These dialysis facilities offered free testing to measure anti-S IgG titers for HD patients and HCWs as a health check-up service, as previously described.[7] We enrolled 798 participants, divided into two groups, one with HD three times a week (i.e., the HD group) and a control group of HCWs (i.e., the HCW group). All participants were aged > 20 years and received two doses of the BNT162b2 vaccine between May and August 2021. The third dose of the BNT162b2 vaccine was administered 6 months after the second dose, according to the Japanese government's COVID-19 vaccination strategy. All participants were vaccinated following the vaccination schedule as per the manufacturer's instructions (BNT162b2, Comirnaty®, Pfizer–BioNTech).

Eighteen participants with documented SARS-CoV-2 infection before or during the study period were excluded. We also excluded 300 participants: 1) who had been treated for cancer within the past 1 year; 2) had taken steroids or immunosuppressants within the past 1 year; 3) had undergone peritoneal dialysis or renal transplantation; 4) had been diagnosed with immunodeficiency syndrome; 5) had missing records. Finally, we selected 350 patients on HD and 130 HCWs. Clinical data, dialysis schedules, and anti-S-IgG titer values were obtained from medical records.

Anti-S IgG titer estimation

The titers of anti-S IgG were serologically measured at 1 and 6 months after the second dose of vaccine and 1 month after the third dose in the HD and HCW groups. The titers at 6 months after the second dose in both groups were measured immediately before the third dose of the vaccine. We performed the Abbott Architect SARS-CoV-2 IgG II Quant chemiluminescent microparticle immunoassay to detect IgG antibodies in the receptor-binding domain of the S1 subunit of the SARS-CoV-2 spike protein S1. As per the manufacturer's instructions, the results are presented as AU/ml, with a cut-off value for positive results (responders) of ≥ 50 AU/ml. The reportable measurement range of the assay was 6.8–80,000 AU/ml. A range > 10,000 AU/ml is reported in thousands of

units. Regarding within-laboratory precision, the coefficient of variation was reported to be between 4.2% and 5.1%.[S16] Levels below the detection limit for the antibody were considered equivalent to 6.79 AU/ml in this study because the detection limit was 6.8 AU/ml.

Definitions of response status after vaccination

All participants were defined on the basis of the level of anti-S IgG antibodies measured after the vaccine dose: no responders, low responders, and high responders. Maxime et al. reported that a titer of anti-S IgG ≥ 997 BAU/ml was associated with the viral neutralizing capacity of the serum.[S11] They defined “high” (anti-S IgG ≥ 997 BAU/ml) and “low or no” (anti-S IgG < 997 BAU/ml) responses to the vaccine.[S11] The mathematical relationship between the Abbott AU/ml unit and the WHO BAU/ml unit follows the equation BAU/ml = 0.142 × AU/ml.[S17] A value of 997 BAU/ml is equivalent to 7021 AU/ml. The response status was defined as follows: no response to the vaccine (anti-S IgG < 50 AU/ml), low response (50–7021 AU/ml), and high response (≥ 7021 AU/ml).

Statistical analysis

Categorical variables were expressed as percentages and compared using the chi-squared test or Fisher's exact test. Continuous variables were expressed as mean ± SD and compared using Welch's *t*–test or analysis of covariance to detect the difference in log-transformed anti-S IgG titers at each time point with adjustment for age, sex, and body mass index, which were only factors retrospectively available in both groups that have significant impact on the anti-S IgG titers after SARS-CoV-2 mRNA vaccination,[S18-S20] or as median and IQR and compared using the Mann–Whitney U-test or Kruskal–Wallis test for variables with non-normal distribution. The results were considered statistically significant if the P-value was < 0.05. SPSS Statistics for Windows version 22 (IBM Corp., Armonk, N.Y., USA) was used for statistical analyses.

Ethics Statement

This study was conducted in accordance with the principles of the Declaration of Helsinki. The study protocol was approved by the Ethics Committee of Yokohama City University Hospital (IRB approval number: F 220200037).

Informed consent

Because the present study was a retrospective and non-interventional study, we adopted

an opt-out method instead of written informed consent. We provided patients with the opportunity to opt out by explaining the purpose of the study and the required individual data for the study by posting the poster at four dialysis facilities (Kamioooka Jinsei Clinic, Yokohama, Japan; Bunko Jin Clinic, Yokohama; Kanazawa Clinic, Yokohama; and Oppama Jinsei Clinic, Yokosuka, Japan).

Supplementary Results

The final analyses included 350 patients in the HD group (mean age was 72.6 ± 11.5 years, and 29.7% were women) and 130 controls in the HCW group (mean age was 46.1 ± 11.8 years, and 76.9% were women). The clinical history, laboratory data, and dialysis prescription of the HD group are presented in **Supplementary Table S1**. In total, 187 (53.4%) and 169 (48.3%) patients in the HD group had a history of hypertension and diabetes, respectively, and 82.3% had been treated using the online hemodiafiltration method.

The log-transformed anti-S IgG titer in the HD group vs. the HCW group was as follows: mean (95% confidence interval [CI]) 7.69 (95% CI: 7.56 to 7.82) log (AU/ml) vs. 8.85 (95% CI: 8.71 to 8.99) log (AU/ml) at 1 month after the second dose ($P < 0.001$); 5.69 (95% CI: 5.56 to 5.81) vs. 6.79 (95% CI: 6.66 to 6.92) at 6 months after the second

dose ($P < 0.001$); and 9.94 (95% CI: 9.84 to 10.05) vs. 9.94 (95% CI: 9.82 to 10.07) at 1 month after the third dose (**Supplementary Table S2**). In addition, the adjusted mean difference in log-transformed anti-S IgG titers between both groups was estimated by analysis of covariance with adjustment for age, sex, and body mass index as follows: −0.41 (95% CI: −0.78 to −0.04) at 1 month after the second dose ($P < 0.05$), −0.51 (95% CI: −0.86 to −0.16) at 6 months after the second dose ($P < 0.001$), and 0.27 (95% CI: −0.04 to 0.58) at 1 month after the third dose ($P = 0.08$) (**Supplementary Table S2**).

The seronegativity rate of anti-S IgG titers was 1.1% (n = 4) in the HD group and 0% (n = 0) in the HCW group at 1 month after the second dose. However, at 6 months after the second dose, the seronegativity rate of the anti-S IgG titers was significantly higher in HD patients at 7.7% (n = 27) than 0% (n = 0) in the HCW group. One month after the third dose (month 7), the seronegativity rate of anti-S IgG was 0% (n = 0) in both groups (**Supplementary Table S3**).

Furthermore, we evaluated the antibody responses to the second and third doses in the HD and HCW groups. The composite outcome (defined as the cumulative percentage of patients switching from their original response status at 1 month after the second dose to one-step higher or more status at 1 month after the third dose, i.e., from no to low response status, from no to high response status, and from low to high

response status) was achieved in 86.8% (262 of 302) in the HD group ($P < 0.001$, Bowker's test). In detail, 100% (4 of 4) changed from no to low response status, 0% (none of 4) from no to high response status, 86.6% (258 of 298) from low to high response status (**Supplementary Table S4**). In the HCW group, 91.2% (52 of 57) changed from low response status at 1 month after the second dose to high response status at 1 month after the third dose ($P < 0.001$, McNemar's test) (**Supplementary Table S5**).

Table S1. Characteristics of participants at baseline

	Overall (n = 480)	HD group (n = 350)	HCW group (n = 130)	*P*–value†
Age, years	65.4 ± 15.5	72.6 ± 11.5	46.1 ± 11.8	< 0.001
Women, n (%)	204 (42.5)	104 (29.7)	100 (76.9)	< 0.001
Body mass index, kg/m^2‡	22.4 ± 4.1	22.6 ± 4.2	22.0 ± 3.6	0.149
Hypertension, n (%)	198 (41.3)	187 (53.4)	11 (8.5)	< 0.001
Diabetes, n (%)	171 (35.6)	169 (48.3)	2 (1.5)	< 0.001
Dyslipidemia, n (%)	48 (10.0)	37 (10.6)	11 (8.5)	0.608
Cardiovascular disease, n (%)	192 (40.0)	191 (54.6)	1 (0.8)	< 0.001
**Clinical data and prescription of HD in the HD group (n = 350)**				
Cause of HD				
Diabetes, n (%)		141 (40.3)		
Hypertension, n (%)		69 (19.7)		
Glomerular nephritis, n (%)		92 (26.3)		
Others or unknown, n (%)		51 (14.6)		
Type of dialysis				
Hemodialysis, n (%)		62 (17.7)		
Predilution on-line HDF, n (%)		288 (82.3)		
Vascular access				
Arteriovenous fistula, n (%)		329 (94.0)		
Arteriovenous graft, n (%)		18 (5.1)		
Others, n (%)		3 (0.9)		

Duration of dialysis, years	4.0 (1.8–9.3)
Kt/V urea, per week	1.3 (1.1–1.4)
Time of dialysis at once, hours	4.0 (3.5–4.0)
Substitution volume in HDF, L/session	36.0 (24.0–42.0)
Hemoglobin level, g/dL	11.3 ± 1.1
Total protein level, g/dL	6.5 ± 0.5
Albumin level, g/dL	3.7 ± 0.4
Use of vitamin D	200 (57.1)

Data as of the first dose of vaccine were collected as baseline. They are expressed as mean ± standard deviation, median (quartiles) for skewed variables, and counts (percentages). †HD group vs. HCW group via Welch's *t*-test, the chi-squared test or Fisher's exact test where appropriate. ‡Dry weight was used as body weight in the HD group. HCW, healthcare worker; HD: hemodialysis; HDF: hemodiafiltration.

Table S2. Titers of anti-S IgG after the second and third vaccination doses

Measurement	HD group n = 350		HCW group n = 130		Adjusted mean difference in log-transformed values
	Absolute values	Log-transformed	Absolute values	Log-transformed	
**1 month after the second dose**	2538.8 (1185.6–4938.1)	7.69 (7.56 to 7.82)	7645.1 (4856.8–11,000)	8.85 (8.71 to 8.99)	−0.41* (−0.78 to −0.04)
**6 months after the second dose**	312.8 (157.9–613.6)	5.69 (5.56 to 5.81)	803.8 (498.4–1342.7)	6.79 (6.66 to 6.92)	−0.51** (−0.86 to −0.16)
**1 month after the third dose**	24,500 (11,000–40,000)	9.94 (9.84 to 10.05)	20,000 (12,750–32,250)	9.94 (9.82 to 10.07)	0.27 (−0.04 to 0.58)

The antibody titers at 1 month and 6 months after the second dose and at 1 month after the third dose are shown in absolute values (median (quartiles)) and in natural logarithm form (mean [95% confidence interval]). The interval between the second and third doses was 6 months. The measurement at 6 months after the second dose was performed just before the third dose.

Adjusted mean difference was estimated by analysis of covariance with adjustment for age, sex, and body mass index. *$P < 0.05$, **$P < 0.01$.

Table S3. The seronegativity rate of SARS-CoV-2 spike protein antibody after the second dose

**Seronegativity rate (<50 AU/ml)**	**HD group n = 350**	**HCW group n = 130**	***P*-value†**
**1 month after the second dose**	1.1% (n = 4)	0% (n = 0)	0.58
**6 months after the second dose**	7.7% (n = 27)	0% (n = 0)	< 0.001
**1 month after the third dose**	0% (n = 0)	0% (n = 0)	-

The seronegativity rates of SARS-CoV-2 spike protein antibody at 1 month and 6 months after the second dose and 1 month after the third dose are shown. The interval between the second and third doses was 6 months. The measurement at 6 months after the second dose was performed just before the third dose. The threshold for the negativity was defined as <50 AU/ml. †HD group vs. HCW group via Fisher's exact test.

Table S4. Number of each response status group between the second and third doses of BNT162b2 in the HD group

HD group (n = 350)		1 month after the third dose (Responder status after three doses) No-responder <50 AU/ml	Low-responder 50–7021 AU/ml	High-responder ≥7021 AU/ml	Sub total
1 month after the second dose (Responder status after two doses)	No-responder <50 AU/ml	0	4	0	4
	Low-responder 50–7021 AU/ml	0	40	258	298
	High-responder ≥7021 AU/ml	0	0	48	48
Sub total		0	44	306	350

The responder status after the second and third doses is shown. The interval between the second and third doses was 6 months. No response was defined as levels <50 AU/ml, low response as levels between 50 and 7021 AU/ml, and high response as levels ≥7021 AU/ml. Bowker's test for symmetry was performed, $P < 0.001$.

Table S5. Number of each responder status group between the second and third doses of BNT162b2 in the HCW group

HCW group (n = 130)		1 month after the third dose (Responder status after three injections)			Sub total
		No-responder <50 AU/ml	Low-responder 50–7021 AU/ml	High-responder ≥7021 AU/ml	
1 month after the second dose (Responder status after two doses)	No-responder <50 AU/ml	0	0	0	0
	Low-responder 50–7021 AU/ml	0	5	52	57
	High-responder ≥7021 AU/ml	0	0	73	73
Sub total		0	5	125	130

The responder status after the second and third doses is shown. The interval between the second and third doses was 6 months. No response was defined as levels <50 AU/ml, low response as levels between 50 and 7021 AU/ml, and high response as levels ≥7021 AU/ml. McNemar's test for symmetry was performed, $P < 0.001$.

Supplementary References

S1. Kato S, Chmielewski M, Honda H, et al. Aspects of immune dysfunction in end-stage renal disease. *Clin J Am Soc Nephrol*. 2008;3:1526–1533. doi: 10.2215/CJN.00950208.

S2. Betjes MG. Immune cell dysfunction and inflammation in end-stage renal disease. *Nat Rev Nephrol*. 2013;9:255–265. doi: 10.1038/nrneph.2013.44.

S3. Chen CC, Koenig A, Saison C, et al. CD4+ T cell help is mandatory for naive and memory donor-specific antibody responses: Impact of therapeutic immunosuppression. *Front Immunol*. 2018;9:275. doi: 10.3389/fimmu.2018.00275.

S4. Espi M, Koppe L, Fouque D, et al. Chronic kidney disease-associated immune dysfunctions: Impact of protein-bound uremic retention solutes on immune cells. *Toxins*. 2020;12:300. doi: 10.3390/toxins12050300.

S5. Masakane I, Sakurai K. Current approaches to middle molecule removal: room for innovation. *Nephrol Dial Transplant*. 2018;33(suppl_3):iii12-iii21. doi: 10.1093/ndt/gfy224.

S6. Nongnuch A, Ngampongpan W, Srichatrapimuk S, et al. Immune response to influenza vaccination in ESRD patients undergoing hemodialysis vs. hemodiafiltration. *PLoS One*. 2020;15(2):e0227719. doi: 10.1371/journal.pone.0227719.

S7. Yanay NB, Freiman S, Shapira M, et al. Experience with SARS-CoV-2 BNT162b2 mRNA vaccine in dialysis patients. *Kidney Int*. 2021;99:1496–1498. doi: 10.1016/j.kint.2021.04.006.

S8. Angel-Korman A, Peres E, Bryk G, et al. Diminished and waning immunity to COVID-19 vaccination among hemodialysis patients in Israel: The case for a third vaccine dose. *Clin Kidney J*. 2022;15:226–234. doi: 10.1093/ckj/sfab206.

S9. Nordström P, Ballin M, Nordström A, et al. Risk of infection, hospitalisation, and death up to 9 months after a second dose of COVID-19 vaccine: a retrospective, total population cohort study in Sweden. *Lancet*. 2022;399:814–823. doi: 10.1016/S0140-6736(22)00089-7.

S10. Stervbo U, Blazquez-Navarro A, Blanco EV, et al. Improved cellular and humoral immunity upon a second BNT162b2 and mRNA-1273 boost in prime-boost vaccination no/low responders with end-stage renal disease. *Kidney Int*. 2021;100:1335–1337. doi: 10.1016/j.kint.2021.09.015.

S11. Espi M, Charmetant X, Barba T, et al. A prospective observational study for justification, safety, and efficacy of a third dose of mRNA vaccine in patients receiving maintenance hemodialysis. *Kidney Int*. 2022;101:390–402. doi: 10.1016/j.kint.2021.10.040.

S12. Simon B, Rubey H, Gromann M, et al. SARS-CoV-2 antibody and T cell response after a third vaccine dose in hemodialysis patients compared with healthy controls. *Vaccines*. 2022;10:694. doi: 10.3390/vaccines10050694.

S13. Jeulin H, Labat C, Duarte K, et al. Anti-spike IgG antibody kinetics following the second and third doses of BNT162b2 vaccine in nursing home residents. *J Am Geriatr Soc*. 2022. doi: 10.1111/jgs.17837.

S14. Eliakim-Raz N, Leibovici-Weisman Y, Stemmer A, et al. Antibody titers before and after a third dose of the SARS-CoV-2 BNT162b2 vaccine in adults aged ≥60 years. *JAMA*. 2021;326:2203–2204. doi: 10.1001/jama.2021.19885.

S15. Regev-Yochay G, Gonen T, Gilboa M, et al. Efficacy of a fourth dose of Covid-19 mRNA vaccine against omicron. *N Engl J Med*. 2022;386:1377–1380. doi: 10.1056/NEJMc2202542.

S16. Abbott Core Laboratory. SARS-CoV-2 immunoassays: Advancing diagnostics of COVID-19. https://www.corelaboratory.abbott/int/en/offerings/segments/infectious-disease/sars-cov-2. Accessed March 8, 2022.

S17. Mattiuzzo G, Bentley EM, Hassall M, et al. Establishment of the WHO International Standard and Reference Panel for anti-SARS-CoV-2 antibody. https://www.who.int/publications/m/item/WHO-BS-2020.2403. Accessed December 12, 2021.

S18. Collier D A, Ferreira I, Kotagiri P, et al. Age-related immune response heterogeneity to SARS-CoV-2 vaccine BNT162b2. Nature. 2021;596:417–422. doi: 10.1038/s41586-021-03739-1.

S19. Demonbreun A R, Sancilio A, Velez M E, et al. COVID-19 mRNA Vaccination Generates Greater Immunoglobulin G Levels in Women Compared to Men. *J Infect Dis*. 2021;224:793–797. doi: 10.1093/infdis/jiab314.

S20. Zhang H, Liu X, Liu Q, et al. Serological reactivity of inactivated SARS-CoV-2 vaccine based on an S-RBD neutralizing antibody assay. *Int J Infect Dis*. 2022;117:169–173. doi: 10.1016/j.ijid.2022.01.064.
